# Supplementary material for: Ultraconserved elements (UCEs) illuminate the population genomics of a recent, high-latitude avian speciation event
Source: PeerJ. 2018 Oct 5;6:e5735. doi: 10.7717/peerj.5735 (PMC6174879; doi:10.7717/peerj.5735)
Supplement: Supplemental Information 1 — Table S1, Figures S1–S4, loci with high Fst. [file peerj-06-5735-s001.docx]

**Supplemental Information**

**Table S1.** Museum specimens used in this study. All are from the University of Alaska Museum (UAM). Sequences are deposited in NCBI SRA accession SRP151072.

| **UAM #** | **Taxon** | **Age** | **Sex** | **Date** | **Locality** | **Field #** | **SRA #** |
| --- | --- | --- | --- | --- | --- | --- | --- |
| 7066 | *Plectrophenax hyperboreus* | AD | M | 25-Jul-97 | USA: Alaska: Bering Sea, St. Matthew Island | KSW1610 | SAMN09469434 |
| 7407 | *Plectrophenax hyperboreus* | AHY | F | 24-Jul-97 | USA: Alaska: Bering Sea, St. Matthew Island | KSW1588 | SAMN09469433 |
| 7524 | *Plectrophenax hyperboreus* | AD | F | 25-Jul-97 | USA: Alaska: Bering Sea, St. Matthew Island | KSW1611 | SAMN09469435 |
| 8200 | *Plectrophenax hyperboreus* | AD | F | 25-Jul-97 | USA: Alaska: Bering Sea, St. Matthew Island | KSW1617 | SAMN09469436 |
| 8474 | *Plectrophenax nivalis* | U | M | 22-Sep-98 | USA: Alaska: Alaska Peninsula, Cold Bay, Frosty Peak | KSW2691 | SAMN09469440 |
| 8476 | *Plectrophenax nivalis* | IM | F | 22-Sep-98 | USA: Alaska: Alaska Peninsula, Cold Bay, Frosty Peak | KSW2690 | SAMN09469439 |
| 11856 | *Plectrophenax nivalis* | AHY | F | 29-Jun-99 | USA: Alaska: Alaska Peninsula, Cold Bay, Frosty Peak | CLP163 | SAMN09469437 |
| 27725 | *Plectrophenax nivalis* | AD | M | 5-Jun-09 | USA: Alaska, Aleutian Islands, Kiska Island | JJW292 | SAMN09469438 |

**Supplemental Information, cont.**

**
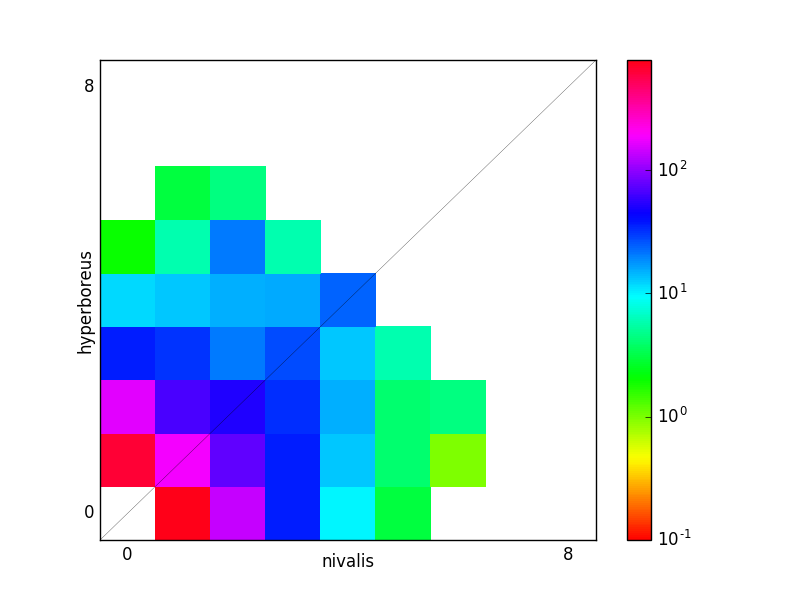
**

**Figure S1**. A 2D FS plot of the folded spectrum analyzed using δaδi, in which the color scale represents the logarithm of the number of variants (Gutenkunst et al. 2009).

**Figure S2**. Frequency distribution of locus lengths.

**Figure S3a**. Distribution of *F_ST_* values among SNP sites between *Plectrophenax* *hyperboreus* and *P. nivalis* (excludes 7,662 sites with *F_ST_* = 0).

**Figure S3b**. Distribution of *F_ST_* values among 521 loci between *Plectrophenax* *hyperboreus* and *P.* *nivalis* (excludes 2,113 loci with *F_ST_* = 0).


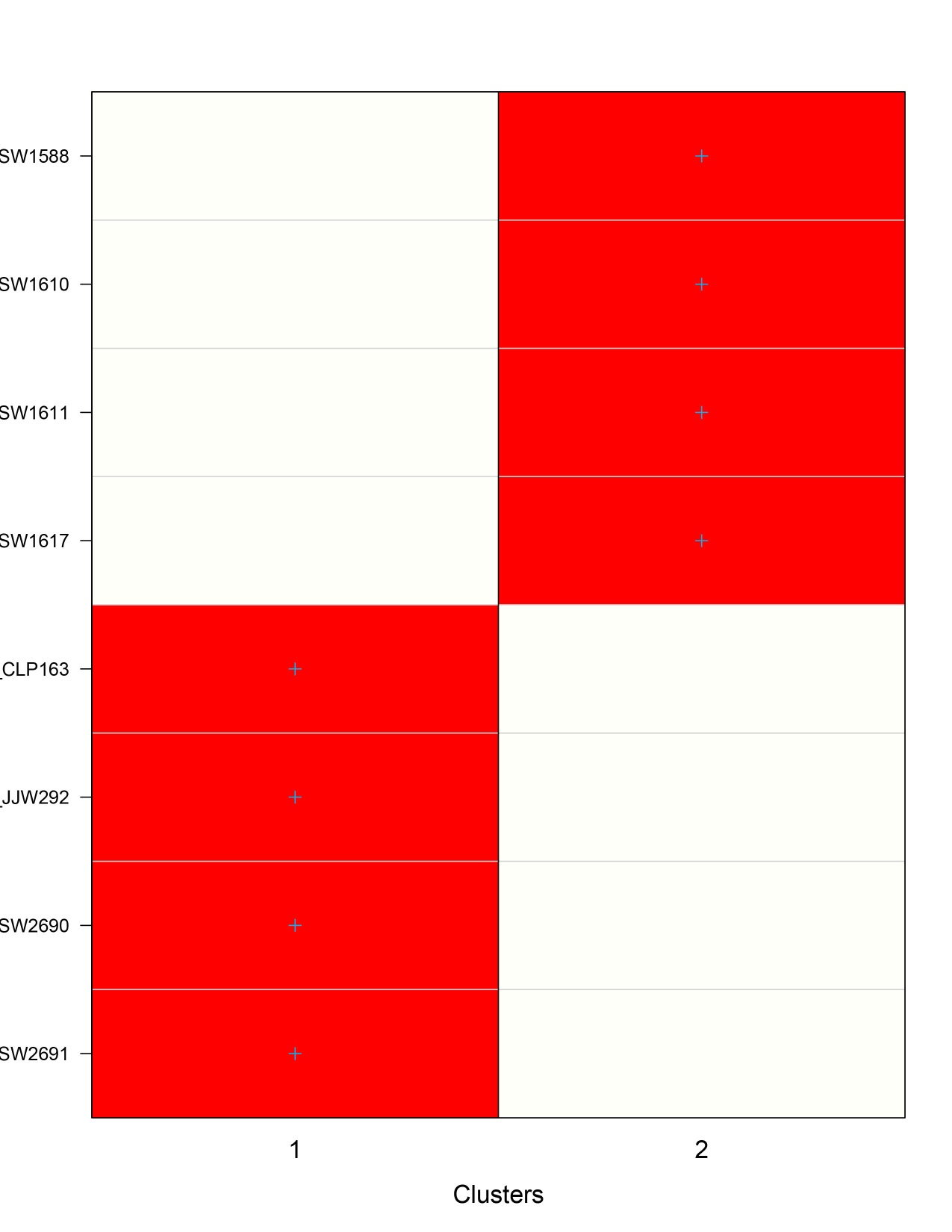


K

K

K

K

K

K

**Figure S4**. Group assignments using Discriminant Analysis of Principal Components (DAPC) in adegenet (Jombart & Ahmed 2011). Cluster 1 is *P. nivalis*; cluster 2 is *P. hyperboreus*.

**Supplemental Information, cont.**

The five bunting loci with the highest *F_ST_* values and the best BLAST match with the zebra finch genome:

**Query= uce-5659_ALL_buntings_assembled_contigs |uce-5659**

Length=1087

Score E

Sequences producing significant alignments: (Bits) Value

ref|NC_011475.1| Taeniopygia guttata isolate Black17 chromoso... 1869 0.0

ALIGNMENTS

>ref|NC_011475.1| Taeniopygia guttata isolate Black17 chromosome 11, Taeniopygia_guttata-3.2.4

Length=21403021

Features flanking this part of subject sequence:

292623 bp at 5' side: LOW QUALITY PROTEIN: iroquois-class homeodomain protein I...

223208 bp at 3' side: alpha-ketoglutarate-dependent dioxygenase FTO

Score = 1869 bits (1012), Expect = 0.0

Identities = 1063/1088 (98%), Gaps = 1/1088 (0%)

**Query= uce-2530_ALL_buntings_assembled_contigs |uce-2530**

Length=1104

Score E

Sequences producing significant alignments: (Bits) Value

ref|NC_011468.1| Taeniopygia guttata isolate Black17 chromoso... 1709 0.0

ALIGNMENTS

>ref|NC_011468.1| Taeniopygia guttata isolate Black17 chromosome 4A, Taeniopygia_guttata-3.2.4

Length=20704505

Features in this part of subject sequence:

dachshund homolog 2 isoform X3

dachshund homolog 2 isoform X1

Score = 1709 bits (925), Expect = 0.0

Identities = 1053/1112 (95%), Gaps = 20/1112 (2%)

**Query= uce-3157_ALL_buntings_assembled_contigs |uce-3157**

Length=1058

Score E

Sequences producing significant alignments: (Bits) Value

ref|NC_011466.1| Taeniopygia guttata isolate Black17 chromoso... 1491 0.0

ALIGNMENTS

>ref|NC_011466.1| Taeniopygia guttata isolate Black17 chromosome 3, Taeniopygia_guttata-3.2.4

Length=112617285

Features flanking this part of subject sequence:

25635 bp at 5' side: parkin coregulated gene protein

23761 bp at 3' side: protein quaking

Score = 1491 bits (807), Expect = 0.0

Identities = 1000/1087 (92%), Gaps = 37/1087 (3%)

**Query= uce-1915_ALL_buntings_assembled_contigs |uce-1915**

Length=1038

Score E

Sequences producing significant alignments: (Bits) Value

ref|NC_011493.1| Taeniopygia guttata isolate Black17 chromoso... 1541 0.0

ALIGNMENTS

>ref|NC_011493.1| Taeniopygia guttata isolate Black17 chromosome Z, Taeniopygia_guttata-3.2.4

Length=72861351

Features in this part of subject sequence:

LOW QUALITY PROTEIN: doublesex- and mab-3-related transcr...

Score = 1541 bits (834), Expect = 0.0

Identities = 984/1051 (94%), Gaps = 32/1051 (3%)

**Query= uce-451_ALL_buntings_assembled_contigs |uce-451**

Length=1206

Score E

Sequences producing significant alignments: (Bits) Value

ref|NC_011465.1| Taeniopygia guttata isolate Black17 chromoso... 1971 0.0

ALIGNMENTS

>ref|NC_011465.1| Taeniopygia guttata isolate Black17 chromosome 2, Taeniopygia_guttata-3.2.4

Length=156412533

Features flanking this part of subject sequence:

419746 bp at 5' side: hepatocyte nuclear factor 4-gamma

138007 bp at 3' side: zinc finger homeobox protein 4

Score = 1971 bits (1067), Expect = 0.0

Identities = 1164/1208 (96%), Gaps = 18/1208 (1%)
